# Burnout and well-being in obstetricians and gynaecologists in Hong Kong: a territory-wide cross-sectional survey

**DOI:** 10.3389/fpubh.2026.1814810

**Published:** 2026-07-22

**Authors:** Tsz Wing Chau, Jessica Yun Pui Law, Wing Cheong Leung, Karen Kar Loen Chan, Yi Man Isabella Wah, Karen Ng, Ho Sze Jacqueline Lee

**Affiliations:** 1 Department of Obstetrics and Gynaecology, Prince of Wales Hospital, Hong Kong, Hong Kong SAR, China; 2 Department of Obstetrics and Gynaecology, Pamela Youde Nethersole Eastern Hospital, Hong Kong, Hong Kong SAR, China; 3 Department of Obstetrics and Gynaecology, Kwong Wah Hospital, Hong Kong, Hong Kong SAR, China; 4 Department of Obstetrics and Gynaecology, The University of Hong Kong, Hong Kong, Hong Kong SAR, China

**Keywords:** anxiety, burnout - professional, depression, doctor, Hong Kong, obstetrics and gynaecology, occupational health, suicidal ideation

## Abstract

This study aims to assess the prevalence and severity of burnout, depression, suicidal ideation, and anxiety among Obstetrics and Gynaecology (OB-GYN) doctors in Hong Kong and identify their contributing factors. All OB-GYN trainees and specialists in Hong Kong were invited to complete a voluntary online cross-sectional survey. This survey used standardized questionnaires including Copenhagen Burnout Inventory (CBI) for burnout, Patient Health Questionnaire-9 (PHQ-9) for depression, and Generalized Anxiety Disorder-7 (GAD-7) for anxiety. A total of 218 doctors completed the survey. Overall, 43.1% of respondents perceived the well-being status of OB-GYN doctors as “poor”; 36.1% as “average” and 20.8% as “excellent.” Using a CBI subscale cut-off score of ≥50 (moderate and higher), 56.4% reported personal burnout; 52.8% reported work-related burnout; and 45.4% reported client-related burnout. The prevalence of depression was 21.1% with 13.3% reported presence of suicidal ideation. The prevalence of anxiety was 20.2%. The most cited sources of stress were work demands, followed by working hours and on-call duties. Residents-in-training and those worked more than 50 h a week had higher prevalence of burnout, suicidal ideation and anxiety while having children had protective effect on doctors’ well-being. Possible interventions including limiting excessive work hours and on-call duties, promoting flexible work arrangements, protected training activities, reviewing training curriculum and streamlining the logbook, dedicated mental health and wellness programs and comprehensive mentorship programs would help to improve the well-being of doctors. This study identified a considerable level of burnout, depression, suicidal ideation and anxiety among OB-GYN doctors especially in residents-in-training and doctors working for more than 50 h a week. Addressing this critical issue will require a collaborative effort involving healthcare organizations, policymakers and mental health professionals.

## 1 Introduction

Over the past decade, there has been an increasing prevalence and publicity of concern on physician well-being and burnout internationally (1). Physician burnout is a long-term stress reaction characterized by depersonalization, including cynical or negative attitudes toward patients, emotional exhaustion, a sense of decreased personal achievement, and a lack of empathy for patients (2). Burnout is included in the 11th Revision of the International Classification of Diseases (ICD-11) as an occupational phenomenon, arising from chronic exposure to occupational stressors.

Physician burnout has been associated with negative personal, clinical, and organizational consequences and outcomes (3). Physician burnout has been reported to be associated with reduced job satisfaction and physical health consequences such as cardiovascular risks, mental health issues, prolonged fatigue, and even occupational injuries (4, 5). One of the major concerns is the impact on the safety and quality of patient care. It has been widely reported that burnout is associated with higher patient safety incidents, poorer doctor-patient relations and higher patient dissatisfaction (6, 7). Burnout is also a significant factor associated with physician attrition (8).

Coupled with the increasing complexity of patients and societal expectations, Obstetrics and Gynaecology (OB-GYN) is notorious for high levels of physician burnout and trainee attrition due to the demanding nature of their profession (9). In a National Survey the United States of more than 9,000 physicians in over 29 specialties (10), 49% of physicians reported they were burned out and OB-GYN was ranked 2nd in the highest rate among specialties. According to a Royal College of Obstetricians and Gynaecologists report (11), attrition rates amongst trainees are approaching 30%- one the highest in any specialty.

In Hong Kong, OB-GYN services are delivered through a dual system of public hospitals under the Hospital Authority and a smaller but important private sector that provides mainly elective maternity and gynaecological care. Public OB-GYN units are characterized by heavy caseloads, 24-h emergency coverage and training responsibilities, with doctors often managing complex, high-risk pregnancies and emergency gynaecological conditions within resource-constrained environments. On the other hand, private OB-GYN practice typically involves lower patient volumes, more elective work, greater control over scheduling and higher remuneration, but exposure to market pressures and medicolegal concerns associated with fee-for-service care. These challenges can potentially contribute to stress, burnout, and reduced quality of life among Obstetricians and Gynaecologists. The attrition rate of full-time OB-GYN doctors in public hospitals in 2019 was reported to be 9.2%, ranking third among all specialties (12). On an organizational level, an increased attrition potentially has a large economic cost to the system related to reduced productivity related to physician burnout and attrition (13).

There is a limited body of research exploring the issues of burnout and well-being among OB-GYN doctors in Hong Kong. A previous cross-sectional survey evaluated the burnout and health status burnout among young doctors in the region (14), but it only included 19 OB-GYN doctors and did not capture a substantial proportion of doctors working in the private sector.

In line with the Hong Kong Academy of Medicine’s Well Being Initiative (15), this study aimed to evaluate the prevalence of burnout and well-being of the OB-GYN workforce in Hong Kong, as well as the factors that contribute to their well-being. The findings of this research hold the potential to inform policy makers, healthcare institutions, and professional bodies in Hong Kong regarding the development and implementation of strategies aimed at improving the well-being of obstetricians and gynaecologists. This study may contribute to the global body of knowledge on healthcare provider well-being, providing valuable insights that can be applied to other regions facing similar challenges.

## 2 Methods

### 2.1 Survey design

In this study, all Hong Kong OB-GYN trainees and specialists registered with the Hong Kong College of Obstetricians and Gynaecologists were invited to complete an anonymous voluntary cross-sectional electronic survey between 01/06/2023 and 30/05/2024. The survey consisted of self-reported demographic data, years of experience in OB-GYN, and current professional details. Burnout was assessed using the Copenhagen Burnout Inventory (CBI). In addition, as depression and anxiety represent specific mental health outcomes that are closely associated with impaired well-being and frequently co-occur with burnout, yet remain distinct clinical entities with important implications for functioning, quality of life, and risk of self-harm (16, 17), the survey also included comprehensive assessment of symptoms of depression and anxiety using the Patient Health Questionnaire-9 (PHQ-9) and the Generalized Anxiety Disorder-7 (GAD-7) respectively. The survey selected validated and widely used screening instruments to facilitate comparison with existing literature and provide clinically relevant findings that could inform targeted interventions.

#### 2.1.1 Copenhagen Burnout Inventory

This tool consists of three distinct scales designed to measure personal burnout, work-related burnout, and client-related burnout. The personal burnout scale gauges the overall degree of fatigue experienced by the respondent, irrespective of their work experience. The work-related burnout scale measures the level of fatigue specifically related to the individual’s work, exploring how their perception of their job and work environment contributes to feelings of exhaustion. The client-related burnout scale assesses the perceived degree of fatigue associated with working directly with patients. The burnout level is calculated as a mean score across these three scales, with each scale having a value ranging from 0 to 100. A score of 50 or higher on any of the scales indicates a high degree of burnout. All items are straightforward, positively skewed, relate to the relevant subscale and have high internal reliability (18). In the current study, Cronbach alpha reliability coefficients of the CBI subscales were excellent (personal Cronbach’s *α* = 0.94; work-related Cronbach’s α = 0.94; and client-related Cronbach’s *α* = 0.93).

#### 2.1.2 Patient Health Questionnaire-9

The PHQ-9 is a depression assessment instrument that evaluates the presence and severity of depressive symptoms. This tool measures each of the nine criteria for depression specified in the Diagnostic and Statistical Manual of Mental Disorders, Fourth Edition (DSM-IV), using a scale ranging from “0” (not at all) to “3” (nearly every day). A PHQ-9 score greater than 9 has been reported to have a sensitivity of 88% and a specificity of 88% for major depressive episodes (19). In the current study, the internal consistency was excellent (Cronbach’s *α* = 0.92).

#### 2.1.3 Generalized Anxiety Disorder-7

The GAD-7 is an anxiety assessment tool, developed based on Diagnostic and Statistical Manual of Mental Disorders IV criteria for identifying likely cases of generalized anxiety disorder, on a scale ranging from “0” (not at all) to “3” (nearly every day). A GAD-7 score greater than 9 has a reported sensitivity of 89% and a specificity of 82% for GAD (20). In the current study, the internal consistency was excellent (Cronbach’s *α* = 0.94).

#### 2.1.4 Overall well-being, stressors and suggested interventions

The final section of the survey, titled “How can we help?,” focused on participants’ perceptions of the overall well-being, their main sources of stress, and potential measures to improve well-being. First, practitioners were asked about their perceptions of the overall well-being of Obstetricians and Gynaecologists in Hong Kong with the question: “What is your opinion on the current well-being status of OB-GYN doctors in Hong Kong?,” rated on a 5-point Likert scale from 1 (“poor”) to 5 (“excellent”), with 3 representing “average” well-being.

Next, possible sources of stress including “work demand,” “working hours,” “on-call duties,” “workplace relationship,” “examination,” “family,” “personal relationship,” “finance,” “health” and “spirituality,” were ranked from 1st to 10th to investigate factors contributing to doctors’ burnout.

The survey ended with an open-ended question on “What can College do to improve well-being” which allowed participants to provide free-text responses to generate concrete and comprehensive suggestions about possible measures to improve the well-being of OB-GYN doctors.

### 2.2 Study population

The online survey was developed and distributed to all registered OB-GYN trainees and specialists in Hong Kong in electronic format. The doctors who completed the survey were asked to refer the survey to their colleagues who have not yet completed the survey. The invitations to participate were also sent via email. The study protocol complied with the Declaration of Helsinki and was approved by The Joint Chinese University of Hong Kong-New Territories East Cluster Clinical Research Ethics Committee (Reference number: 2023.204).

The survey began with an information page explaining the study purpose, procedures, potential risks and benefits, confidentiality measures, and participants’ right to withdraw at any time without penalty. Participants provided informed consent by selecting “Agree” before accessing the survey questions.

Patients and the public were not involved in any way.

### 2.3 Sample size calculation

Raosoft software was used for sample size calculation (Raosoft Inc., Seattle, WA, United States).^1^ There are 665 OB-GYN doctors (trainees and specialists included) in Hong Kong. To achieve a 95% confidence interval (CI) with a precision of 5%, 218 participants were required. Our final sample of 218 doctors was sufficient to achieve the desired statistical power.

### 2.4 Statistical analysis

The data were analyzed with software IBM SPSS Version 29.0.1.0. The prevalence of burnout, depression, suicidal ideation and anxiety was shown using point estimates and 95% confidence intervals. Descriptive statistics were presented concerning demographic characteristics. Direct logistic regression analyses were performed to assess the association between demographic and professional characteristics and the likelihood of experiencing personal burnout, work-related burnout, client-related burnout, depression, suicidal ideation, and anxiety. For each outcome, separate models were fitted including gender (male vs. female), marital status (single/separated/divorced vs. married/common law), parental status (without children vs. have children), level of seniority (specialist vs. resident-in-training), setting of practice (public vs. private), and weekly working hours (≤50 vs. > 50 h) as independent variables. Results are presented as odds ratios with 95% confidence intervals and *p* values. The significance level was set at *p* < 0.05.

## 3 Results

### 3.1 Participants demographics

There are a total of 665 OB-GYN trainees and specialists in Hong Kong. All of them were invited and 218 of them completed the survey, yielding a response rate of 32.8%. Among the respondents, 71 (32.6%) were residents-in-training, 73 (33.5%) were specialists working in public sectors and 74 (33.9%) were specialists working in private sectors. During the period when the study was conducted, there was a total of 97 residents-in-training in Hong Kong, and 73.2% (71 out of 97) of them completed the survey. The mean age among all respondents was 40.9 ± 13.1 years. The respondents’ demographic data are summarized in Table 1.

**Table 1:** Respondent demographics (*n* = 218).

Variable	Number (%)
Gender	
Female	160 (73.4%)
Male	54 (24.8%)
Prefer not to answer	4 (1.8%)
Age (years)	40.9 ± 13.1
21–30	63 (28.9%)
31–40	65 (29.8%)
41–50	30 (13.8%)
51–60	30 (13.8%)
61–70	21 (9.6%)
71–80	5 (2.3%)
Prefer not to answer	4 (1.8%)
Marital status	
Married/common law	121 (55.5%)
Single/separated/divorced	90 (41.3%)
Prefer not to answer	7 (3.2%)
Parental status	
No children	120 (55%)
Have children	95 (43.6%)
Prefer not to answer	3 (1.4%)
Current practice	
Residents-in-training	71 (32.6%)
Specialists in public sectors	73 (33.5%)
Specialists in private sectors	74 (33.9%)
Working hours	
</= 30 h	18 (8.3%)
31–40 h	22 (10.1%)
41–50 h	32 (14.7%)
51–60 h	38 (17.4%)
61–70 h	56 (25.7%)
71–80 h	32 (14.7%)
81–90 h	14 (6.4%)
>90 h	6 (2.8%)

### 3.2 General well-being status

Overall, 43.1% (*n* = 93) of respondents perceived the well-being status of OB-GYN doctors in general as “poor,” 36.1% (*n* = 78) perceived the well-being status as “average,” while 20.8% (*n* = 45) perceived the well-being status as “excellent.”

Among residents-in-training, 60.6% (*n* = 43) of respondents perceived the well-being status of OB-GYN doctors as “poor,” 25.4% (*n* = 18) perceived the well-being status as “average,” while 12.7% (*n* = 9) perceived the well-being status as “excellent.” On the other hand, among specialists, 34% (*n* = 50) of respondents perceived the well-being status of OB-GYN doctors as “poor,” 40.8% (*n* = 60) perceived the well-being status as “average,” while 24.5% (*n* = 36) perceived the well-being status as “excellent.”

### 3.3 Prevalence of burnout, depression, suicidal ideation and anxiety

As measured by the CBI, the mean personal burnout score was 54.0 ± 22.9, work-related burnout score was 49.0 ± 24.8, and client-related burnout score was 44.4 ± 23.0. Using a CBI subscale cut-off score of ≥50 (moderate and higher), 56.4% (*n* = 123, 95% CI = 49.6–63.1%) of respondents reported personal burnout; 52.8% (*n* = 115, 95% CI = 45.9–59.5%) of respondents reported work-related burnout; and 45.4% (*n* = 99, 95% CI = 38.7–52.3%) of respondents reported client-related burnout (Table 2).

**Table 2:** General burnout, depression, anxiety (*n* = 218).

Copenhagen Burnout Inventory	Number (%)	95% CI
Personal burnout score	54.0 ± 22.9	
Low (<50)	95 (43.6%)	
Moderate to severe (50–100)	123 (56.4%)	49.6–63.1%
Moderate (50–74)	75 (34.4%)	
High (75–99)	40 (18.3%)	
Severe (100)	8 (3.7%)	
Work-related burnout score	49.0 ± 24.8	
Low (<50)	103 (47.2%)	
Moderate to severe (50–100)	115 (52.8%)	45.9–59.5%
Moderate (50–74)	74 (33.9%)	
High (75–99)	39 (17.9%)	
Severe (100)	2 (0.9%)	
Client-related burnout score	44.4 ± 23.0	
Low (<50)	119 (54.6%)	
Moderate to severe (50–100)	99 (45.4%)	38.7–52.3%
Moderate (50–74)	74 (33.9%)	
High (75–99)	20 (9.2%)	
Severe (100)	5 (2.3%)	
PHQ-9		
None to mild depression (0–9)	172 (78.9%)	
Moderate to severe depression (10–27)	46 (21.1%)	15.9–27.1%
Moderate (10–14)	23 (10.6%)	
Moderately severe (15–19)	11 (5%)	
Severe (20–27)	12 (5.5%)	
Presence of suicidal ideation	29 (13.3%)	9.1–18.5%
GAD-7		
Minimal to mild anxiety (0–9)	174 (79.8%)	
Moderate to severe anxiety (10–21)	44 (20.2%)	15.1–26.1%
Moderate (10–14)	31 (14.2%)	
Severe (15–21)	13 (6%)	

CI, confidence interval.

As measured by the PHQ-9, the prevalence of depression among respondents, defined as a score of ≥10, was 21.1% (*n* = 46, 95% CI = 15.9–27.1%). In total, 13.3% (*n* = 29, 95% CI = 9.1–18.5%) respondents reported thoughts that “wished he or she was dead” or “hurting himself or herself in some way” (Table 2).

As measured by the GAD-7, the prevalence of anxiety among respondents, defined as a score of ≥10, was 20.2% (*n* = 44, 95% CI = 15.1–26.1%; Table 2).

### 3.4 Association of factors for burnout, depression, suicide and anxiety

Logistic regression modelling was performed to investigate bivariate associations of demographic and professional factors with burnout, depression, the presence of suicidal ideation and anxiety.

Residents-in-training (odds ratio [OR] = 2.93; 95% CI = 1.22–7.05; *p* = 0.017; compared with specialists), and those working for more than 50 h per week (OR = 2.17; 95% CI = 1.02–4.60; *p* = 0.044) were associated with higher likelihood of personal burnout. Working for more than 50 h per week (OR = 2.88; 95% CI = 1.31–6.31; *p* = 0.008) was positively associated with work-related burnout. Conversely, having children (OR = 0.35; 95% CI = 0.14–0.89; *p* = 0.028) was associated with lower likelihood of work-related burnout. Having children (OR = 0.28; 95% CI = 0.11–0.69; *p* = 0.006) was also associated with lower likelihood of client-related burnout (Table 3).

**Table 3:** Bivariate logistic model for burnout (CBI score ≥50 compared to <50) (*n* = 206).

Factor	Personal burnout		Work-related burnout		Client-related burnout	
	Odds ratio (95% CI)	*p*-value	Odds ratio (95% CI)	*p*-value	Odds ratio (95% CI)	*p*-value
Gender						
Male	Ref	-	Ref	-	Ref	-
Female	0.95 (0.47–1.93)	0.890	0.77 (0.36–1.61)	0.480	0.72 (0.35–1.49)	0.377
Marital status						
Single/separated/divorced	Ref	-	Ref	-	Ref	-
Married/common law	1.45 (0.59–3.60)	0.418	1.43 (0.57–3.59)	0.441	1.04 (0.44–2.43)	0.932
Parental status						
Without children	Ref	-	Ref	-	Ref	-
Have children	0.42 (0.17–1.03)	0.059	**0.35** (0.14–0.89)	**0.028**	**0.28** (0.11–0.69)	**0.006**
Level of seniority						
Specialists	Ref	-	Ref	-	Ref	-
Residents-in-training	**2.93** (1.22–7.05)	**0.017**	2.05 (0.85–4.92)	0.108	2.09 (0.91–4.84)	0.084
Setting of practice						
Public	Ref	-	Ref	-	Ref	-
Private	1.27 (0.58–2.76)	0.548	0.83 (0.37–1.86)	0.652	1.43 (0.62–3.29)	0.406
Working hours per week						
≤50 h	Ref	-	Ref	-	Ref	-
>50 h	**2.17** (1.02–4.60)	**0.044**	**2.88** (1.31–6.31)	**0.008**	1.45 (0.65–3.24)	0.361

Values in bold are statistically significant (*p* < 0.05).

Residents-in-training (OR = 5.21; 95% CI = 1.21–22.5; *p* = 0.027; compared with specialists) were more likely to report suicidal ideation, whereas female (OR = 0.26; 95% CI = 0.09–0.76; *p* = 0.013) and having children (OR = 0.103; 95% CI = 0.02–0.46; *p* = 0.003) were negatively associated with suicidal ideation (Table 4).

**Table 4:** Bivariate logistic model for depression (PHQ-9 score ≥10 compared to <10), suicidal ideation and anxiety (GAD-7 score ≥10 compared to <10; *n* = 206).

Factor	Depression		Suicidal ideation		Anxiety	
	Odds ratio (95% CI)	*p*-value	Odds ratio (95% CI)	*p*-value	Odds ratio (95% CI)	*p*-value
Gender						
Male	Ref	-	Ref	-	Ref	-
Female	0.82 (0.32–2.10)	0.674	**0.26** (0.09–0.76)	**0.013**	1.82 (0.56–5.91)	0.320
Marital status						
Single/separated/divorced	Ref	-	Ref	-	Ref	-
Married/common law	1.15 (0.42–3.11)	0.787	2.06 (0.66–6.42)	0.212	0.79 (0.26–2.39)	0.674
Parental status						
Without children	Ref	-	Ref	-	Ref	-
Have children	0.31 (0.09–1.07)	0.064	**0.103** (0.02–0.46)	**0.003**	0.43 (0.11–1.77)	0.243
Level of seniority						
Specialists	Ref	-	Ref	-	Ref	-
Residents-in-training	2.48 (0.90–6.88)	0.080	**5.21** (1.21–22.5)	**0.027**	**3.97** (1.24–12.7)	**0.020**
Setting of practice						
Public	Ref	-	Ref	-	Ref	-
Private	1.46 (0.42–5.13)	0.557	2.52 (0.58–10.9)	0.216	1.49 (0.33–6.65)	0.605
Working hours per week						
≤50 h	Ref	-	Ref	-	Ref	-
>50 h	2.17 (0.62–7.60)	0.224	0.407 (0.09–1.78)	0.232	1.63 (0.37–7.10)	0.519

Values in bold are statistically significant (*p* < 0.05).

Residents-in-training (OR = 3.97; 95% CI = 1.24–12.7; *p* = 0.020; compared with specialists) were more likely to have anxiety (Table 4).

### 3.5 Subgroup analysis for burnout, depression, suicide and anxiety

Among residents-in-training, the mean personal burnout score was 66.1 ± 21.5, work-related burnout score was 63.3 ± 22.2, and client-related burnout score was 55.8 ± 24.6 which were much higher than those of specialists (48.1 ± 21.3; 42.1 ± 23.0; 38.9 ± 20.1). Consistent with these mean differences, a markedly greater proportion of residents-in-training fell into the moderate to severe personal burnout range compared with specialists [78.9% (95% CI = 67.6–87.7%) vs. 45.6% (95% CI = 37.4–54%)]. Regarding the distribution of burnout severity, residents-in-training were more likely to fall into the high or severe categories instead of moderate burnout compared to specialists. The presence of suicidal ideation and prevalence of anxiety were also significantly higher in residents-in-training as compared to specialists [19.7% (95% CI = 11.2–30.9%) vs. 10.2% (95% CI = 5.8–16.3%); 36.6% (95% CI = 25.5–48.9%) vs. 12.2% (95% CI = 7.4–18.7%)] (Table 5).

**Table 5:** Burnout, depression, anxiety in residents-in-training compared to specialists.

Copenhagen Burnout Inventory	Residents-in-training (*n* = 71)	Specialists (*n* = 147)
Personal burnout score	66.1 ± 21.5	48.1 ± 21.3
Low (<50)	15 (21.1%)	80 (54.4%)
Moderate to severe (50–100)	**56 (78.9%)** **(95% CI 67.6–87.7%)**	**67 (45.6%)** **(95% CI 37.4–54%)**
Moderate (50–74)	27 (38%)	48 (32.7%)
High (75–99)	23 (32.4%)	17 (11.6%)
Severe (100)	6 (8.5%)	2 (1.4%)
Work-related burnout score	63.3 ± 22.2	42.1 ± 23.0
Low (<50)	17 (23.9%)	86 (58.5%)
Moderate to severe (50–100)	**54 (76.1%)** **(95% CI 64.5–85.4%)**	**61 (41.5%)** **(95% CI 33.4–49.9%)**
Moderate (50–74)	28 (39.4%)	46 (31.3%)
High (75–99)	24 (33.8%)	15 (10.2%)
Severe (100)	2 (2.8%)	0 (0%)
Client-related burnout score	55.8 ± 24.6	38.9 ± 20.1
Low (<50)	25 (35.2%)	94 (63.9%)
Moderate to severe (50–100)	**46 (64.8%)** **(95% CI 52.5–75.8%)**	**53 (36.1%)** **(95% CI 28.3–44.4%)**
Moderate (50–74)	27 (38%)	47 (32%)
High (75–99)	14 (19.7%)	6 (4.1%)
Severe (100)	5 (7%)	0 (0%)
PHQ-9		
None to mild depression (0–9)	46 (64.8%)	126 (85.7%)
Moderate to severe depression (10–27)	**25 (35.2%)** **(95% CI 24.2–47.5%)**	**21 (14.3%)** **(95% CI 9.1–21%)**
Moderate (10–14)	11 (15.5%)	12 (8.2%)
Moderately severe (15–19)	7 (9.9%)	4 (2.7%)
Severe (20–27)	7 (9.9%)	5 (3.4%)
Presence of suicidal ideation	**14 (19.7%)** **(95% CI 11.2–30.9%)**	**15 (10.2%)** **(95% CI 5.8–16.3%)**
GAD-7		
Minimal to mild anxiety (0–9)	45 (63.4%)	129 (87.8%)
Moderate to severe anxiety (10–21)	**26 (36.6%)** **(95% CI 25.5–48.9%)**	**18 (12.2%)** **(95% CI 7.4–18.7%)**
Moderate (10–14)	16 (22.5%)	15 (10.2%)
Severe (15–21)	10 (14.1%)	3 (2%)

Values in bold indicate the categories of well‑being selected as the main focus of comparison between the two groups.

Among doctors working for more than 50 h per week, the mean personal burnout score was 60.7 ± 21.4, work-related burnout score was 57.0 ± 23.0, and client-related burnout score was 49.5 ± 23.1 which were much higher than those working for less than or equal to 50 h per week (40.4 ± 19.7; 32.8 ± 19.9; 34.0 ± 19.1). The prevalence of personal burnout and work-related burnout were significantly higher in those working for more than 50 h per week as compared to those working less [67.8% (95% CI = 59.6–75.3%) vs. 33.3% (95% CI = 22.7–45.4%); 66.4% (95% CI = 58.2–74%) vs. 25% (95% CI = 15.5–36.6%)]. Regarding the distribution of burnout severity, those working more were more likely to fall into the high or severe categories of burnout instead of moderate burnout compared to those working less (Table 6).

**Table 6:** Burnout, depression, anxiety in working hours ≤50 compared to >50.

Copenhagen Burnout Inventory	Working hours > 50 (*n* = 146)	Working hours ≤ 50 (*n* = 72)
Personal burnout score	60.7 ± 21.4	40.4 ± 19.7
Low (<50)	47 (32.2%)	48 (66.7%)
Moderate to severe (50–100)	**99 (67.8%) (95% CI 59.6–75.3%)**	**24 (33.3%) (95% CI 22.7–45.4%)**
Moderate (50–74)	56 (38.4%)	19 (26.4%)
High (75–99)	35 (24%)	5 (6.9%)
Severe (100)	8 (5.5%)	0 (0%)
Work-related burnout score	57.0 ± 23.0	32.8 ± 19.9
Low (<50)	49 (33.6%)	54 (75%)
Moderate to severe (50–100)	**97 (66.4%) (95% CI 58.2–74%)**	**18 (25%) (95% CI 15.5–36.6%)**
Moderate (50–74)	58 (39.7%)	16 (22.2%)
High (75–99)	37 (25.3%)	2 (2.8%)
Severe (100)	2 (1.4%)	0 (0%)
Client-related burnout score	49.5 ± 23.1	34.0 ± 19.1
Low (<50)	67 (45.9%)	52 (72.2%)
Moderate to severe (50–100)	**79 (54.1%) (95% CI 45.7–62.4%)**	**20 (27.8%) (95% CI 17.9–39.6%)**
Moderate (50–74)	55 (37.7%)	19 (26.4%)
High (75–99)	19 (13%)	1 (1.4%)
Severe (100)	5 (3.4%)	0 (0%)
PHQ-9		
None to mild depression (0–9)	106 (72.6%)	66 (91.7%)
Moderate to severe depression (10–27)	**40 (27.4%) (95% CI 20.3–35.4%)**	**6 (8.3%) (95% CI 3.1–17.3%)**
Moderate (10–14)	19 (13%)	4 (5.6%)
Moderately severe (15–19)	10 (6.8%)	1 (1.4%)
Severe (20–27)	11 (7.5%)	1 (1.4%)
Presence of suicidal ideation	**20 (13.7%) (95% CI 8.6–20.4%)**	**9 (12.5%) (95% CI 5.9–22.4%)**
GAD-7		
Minimal to mild anxiety (0–9)	107 (73.3%)	67 (93.1%)
Moderate to severe anxiety (10–21)	**39 (26.7%) (95% CI 19.7–34.7%)**	**5 (6.9%) (95% CI 2.3–15.5%)**
Moderate (10–14)	28 (19.2%)	3 (4.2%)
Severe (15–21)	11 (7.5%)	2 (2.8%)

Values in bold indicate the categories of well‑being selected as the main focus of comparison between the two groups.

Among those with children, the mean personal burnout score was 43.9 ± 19.7, work-related burnout score was 37.1 ± 21.2, and client-related burnout score was 34.0 ± 18.0 which were much lower than those with no children (61.5 ± 22.0; 58.0 ± 23.3; 52.2 ± 23.3). The prevalence of work-related burnout, client-related burnout and presence of suicidal ideation were also significantly lower in those with children as compared to those who do not [32.6% (95% CI = 23.4–43%) vs. 68.3% (95% CI = 59.2–76.5%); 25.3% (95% CI = 16.9–35.2%) vs. 60.8% (95% CI = 51.5–69.6%); 7.4% (95% CI = 3–14.6%) vs. 17.5% (95% CI = 11.2–25.5%)]. Regarding the distribution of burnout severity, those with children were less likely to suffer from high or severe burnout compared to those with no children (Table 7).

**Table 7:** Burnout, depression, anxiety in those having children compared to no children.

Copenhagen Burnout Inventory	Have children (*n* = 95)	No children (*n* = 120)
Personal burnout score	43.9 ± 19.7	61.5 ± 22.0
Low (<50)	58 (61.1%)	36 (30%)
Moderate to severe (50–100)	**37 (38.9%)** **(95% CI 29.1–49.5%)**	**84 (70%)** **(95% CI 61–78%)**
Moderate (50–74)	29 (30.5%)	46 (38.3%)
High (75–99)	8 (8.4%)	31 (25.8%)
Severe (100)	0 (0%)	7 (5.8%)
Work-related burnout score	37.1 ± 21.2	58.0 ± 23.3
Low (<50)	64 (67.4%)	38 (31.7%)
Moderate to severe (50–100)	**31 (32.6%)** **(95% CI 23.4–43%)**	**82 (68.3%)** **(95% CI 59.2–76.5%)**
Moderate (50–74)	25 (26.3%)	48 (40%)
High (75–99)	6 (6.3%)	32 (26.7%)
Severe (100)	0 (0%)	2 (1.7%)
Client-related burnout score	34.0 ± 18.0	52.2 ± 23.3
Low (<50)	71 (74.7%)	47 (39.2%)
Moderate to severe (50–100)	**24 (25.3%)** **(95% CI 16.9–35.2%)**	**73 (60.8%)** **(95% CI 51.5–69.6%)**
Moderate (50–74)	23 (24.2%)	50 (41.7%)
High (75–99)	1 (1.1%)	18 (15%)
Severe (100)	0 (0%)	5 (4.2%)
PHQ-9		
None to mild depression (0–9)	86 (90.5%)	85 (70.8%)
Moderate to severe depression (10–27)	**9 (9.5%) (95% CI 4.4–17.2%)**	**35 (29.2%) (95% CI 21.2–38.2%)**
Moderate (10–14)	3 (3.2%)	19 (15.8%)
Moderately severe (15–19)	4 (4.2%)	7 (5.8%)
Severe (20–27)	2 (2.1%)	9 (7.5%)
Presence of suicidal ideation	**7 (7.4%) (95% CI 3–14.6%)**	**21 (17.5%) (95% CI 11.2–25.5%)**
GAD-7		
Minimal to mild anxiety (0–9)	88 (92.6%)	84 (70%)
Moderate to severe anxiety (10–21)	**7 (7.4%) (95% CI 3–14.6%)**	**36 (30%) (95% CI 22–39%)**
Moderate (10–14)	6 (6.3%)	25 (20.8%)
Severe (15–21)	1 (1.1%)	11 (9.2%)

Values in bold indicate the categories of well‑being selected as the main focus of comparison between the two groups.

### 3.6 Possible interventions

Among the 10 potential stressors, “work demands” were the most frequently cited primary source of stress, followed by “working hours” and “on-call duties.” 79 respondents (36.2%) provided suggestions for measures that The Hong Kong College of Obstetricians and Gynaecologists can implement to improve the well-being of the medical workforce through an open-ended question allowing free-text responses.

The most prominent suggestion, made by 59.5% (47 out of 79) of respondents, was to enhance work-life balance. This included implementing policies that limit excessive work hours and on-call duties, as well as promoting flexible work arrangements, such as part-time options. Additionally, ensuring adequate staffing and effective task delegation could help prevent the overburdening of individual doctors (Table 8).

**Table 8:** Possible interventions.

Category	Examples
Enhancing work-life balance (59.5%, 47 out of 79)	Limit excessive work hours Limit on-call duties Promote flexible work arrangement, e.g., part-time options Ensure adequate staffing Effective task delegation
Enhancing professional development (30.4%, 24 out of 49)	Protected time for training activities Emphasize on qualitative rather than quantitative assessment Streamline the training logbook Offer research collaborations Reward systems
Improving mental health support services (30.4%, 24 out of 49)	Confidential counselling Stress management workshops Peer support groups Mentorship programs Collaborative and supportive work environment
Improving public education and medicolegal support (15.2%, 12 out of 49)	Public awareness campaigns Referral for expert opinion Counselling by senior physicians

The second most common suggestion, made by 30.4% (24 out of 79) of respondents, was enhancing professional development and training opportunities. This involved implementing protected time for training activities without the interference of clinical duties or administrative responsibilities, reviewing the training curriculum and place more emphasis on qualitative aspects rather than simply accumulating a predetermined number of clinical procedures, streamlining the logbook for trainees to reduce the administrative burden, and offering research collaborations. Besides, establishing reward systems that acknowledge the contributions and achievements of OB-GYN doctors could foster a sense of value and appreciation.

Furthermore, 30.4% (24 out of 79) of respondents suggested improving mental health support services. This encompassed confidential counseling, stress management workshops, and peer support groups. Mentorship programs that pair junior doctors with experienced, empathetic mentors could also provide valuable guidance and support. Fostering a collaborative and supportive work environment where doctors feel comfortable discussing challenges and seeking help from their peers and superiors would further contribute to the overall well-being.

Finally, 15.2% (12 out of 79) of respondents suggested the need for improved public education and medicolegal support. This included public awareness campaigns to highlight the unique challenges faced by OB-GYN doctors and the importance of supporting the medical workforce. Providing access to medicolegal support services, such as referral for expert opinion and counselling by senior physicians with similar experiences to provide psychological support, to help doctors navigate complex legal and regulatory issues would also be beneficial.

## 4 Discussion

### 4.1 Main findings

This study aimed to quantify well-being in OB-GYN doctors in Hong Kong; it yielded three main findings. First, there was a high proportion of doctors (43.1%) who viewed the overall well-being of OB-GYN doctor in Hong Kong as “poor.” Second, the self-perceived personal well-being and mental health were worse in residents-in-training than in specialists. The mean burnout score, prevalence of depression and anxiety were high in this group of doctors; mean personal, work-related and client-related scores of ≥50 were observed on the CBI and the prevalence of depression and anxiety were over 30%. Third, the most cited sources of stress were work demands, followed by working hours and on-call duties. Possible interventions to improve the well-being of the medical workforce comprised enhancing work-life balance, reinforcing professional development and training opportunities, improving mental health support services and strengthening public education and medicolegal support.

### 4.2 Well-being of residents-in-training

Burnout among doctors has been acknowledged as a global crisis (1), with its effects manifesting on personal, patient, and institutional levels. As the front-line providers responsible for critical decision-making and hands-on patient management, burnout among trainees can have far-reaching consequences, including more medical errors and lack in quality patient care, poor academic performance on training exams and affecting well-being of peers and patients (21).

Although there have been multiple published reports regarding burnout among doctors (22, 23), territory-wide data focusing on OB-GYN doctors in Hong Kong are lacking. The present study of OB-GYN doctors in Hong Kong revealed concerning levels of burnout among trainees, evidenced by high mean burnout scores (personal 66.1 ± 21.5, work-related 63.3 ± 22.2, client-related 55.8 ± 24.6) in residents-in-training. These were higher than those in the previous study by Kwan et al. (14) (personal 59.6 ± 20.5, work-related 57.3 ± 20.1, client-related 49.0 ± 22.3). The prevalence of depression and anxiety were 35.2 and 36.6% respectively, which were more than triple the previously reported prevalence in general population in Hong Kong (9.8 and 11.1%) (24). These findings align with international studies documenting high rates of burnout, depression, suicidal thought and anxiety among Obstetricians and Gynaecologists (9, 25, 26). These may be attributed to the unpredictable nature of obstetric emergencies, long working hours, frequent on-call duties, emotionally demanding patient interactions (including pregnancy loss and adverse outcomes), and high medicolegal risk. Trainees also need to face steep learning curves with high performance expectations, manage concurrent examination and research requirements, and may have fewer established coping strategies or professional support networks.

### 4.3 Other factors affecting well-being

The study suggested that certain factors may affect the risks of burnout and suicidal ideation. Doctors working for more than 50 h per week were twofold more likely to experience personal burnout (OR = 2.17; 95% CI = 1.02–4.60; *p* = 0.044) and work-related burnout (OR = 2.88; 95% CI = 1.31–6.31; *p* = 0.008). Prolonged working hours lead to sleep deprivation, reduced recovery time, and cumulative fatigue, all of which precipitate burnout and mental health deterioration (27, 28). Observational studies in other settings have similarly shown that extended working hours increase the risk of depressive symptoms, anxiety, and burnout among healthcare professionals (28, 29). Beyond mental health, a WHO/ILO meta-analysis indicated that working at least 55 h per week increases the risk of stroke and ischaemic heart disease (30)^,^ underscoring that policies to limit excessive working hours and protect rest opportunities are likely to benefit both psychological and physical health.

On the other hand, having children appeared to serve as a protective factor. Doctors who had children were less likely to have work-related burnout (OR = 0.35; 95% CI = 0.14–0.89; *p* = 0.028), client-related burnout (OR = 0.28; 95% CI = 0.11–0.69; *p* = 0.006) and suicidal ideation (OR = 0.103; 95% CI = 0.02–0.46; *p* = 0.003). This could be due to greater emotional support and life satisfaction derived from family roles, which can buffer the impact of occupational stress and provide alternative sources of meaning outside work. In Hong Kong, mothers tend to assume a greater share of day-to-day child-rearing and domestic responsibilities, whereas fathers are more commonly positioned as the primary breadwinner. The greater amount of time that mothers spend with their children may facilitate disengagement from clinical responsibilities outside working hours and promote healthier boundaries between work and home life, which could support psychological well-being. At the same time, mothers also face a “double burden” of intensive clinical work alongside substantial caregiving at home. By contrast, fathers may experience stronger pressure to maintain income and career progression, which can limit the time and opportunity to benefit from the potentially protective aspects of family life. To explore how these gendered roles might relate to mental health, stratified analyses were performed to compare the prevalence of burnout, depression, suicidal ideation and anxiety across four groups: men without children, men with children, women without children and women with children (Table 9). For every outcome, both men and women with children were better off than their same-sex peers without children. The absolute gap seemed to be larger for men, particularly for work-related burnout, client-related burnout and depression. On the other hand, the reduction in suicidal ideation associated with having children was especially marked among women. Taken together, these findings suggested that the association between parenthood and mental health might differ by sex, but they did not establish that having children is intrinsically more protective for one sex than the other. It is also possible that physicians with more severe burnout or psychological distress are less likely to form families, so the observed association may partly reflect selection effects rather than a purely causal relationship.

**Table 9:** Mental health outcomes by sex-parenthood group.

Outcome	Men with no children (*n* = 19)	Men with children (*n* = 34)	Women with no children (*n* = 100)	Women with children (*n* = 59)	*p* value
Moderate to severe personal burnout	13 (68.4%)	13 (38.2%)	70 (70%)	23 (39%)	< 0.001
Moderate to severe work-related burnout	16 (84.2%)	9 (26.5%)	65 (65%)	21 (35.6%)	< 0.001
Moderate to severe client-related burnout	15 (78.9%)	8 (23.5%)	58 (58%)	16 (27.1%)	< 0.001
Moderate to severe depression	7 (36.8%)	3 (8.8%)	28 (28%)	5 (8.5%)	0.002
Suicidal ideation	5 (26.3%)	5 (14.7%)	16 (16%)	1 (1.7%)	0.013
Moderate to severe anxiety	4 (21.1%)	2 (5.9%)	32 (32%)	4 (6.8%)	< 0.001

The most cited sources of stress were work demands, followed by working hours and on-call duties. These insights highlighted the importance of addressing workload and work-life balance issues within the healthcare system. Targeted interventions and policies that reduce excessive working hours and promote a healthier work-life integration may be crucial in mitigating the risk of burnout and mental health concerns among this critical segment of the healthcare workforce.

### 4.4 Strengths

To our knowledge, this study, which included 32.8% (218 out of 665) OB-GYN doctors in Hong Kong, represents the most comprehensive survey conducted to date exploring the well-being of OB-GYN doctors in Hong Kong. The results regarding residents-in-training were particularly representative since 73.2% (71 out of 97) of them completed the survey. The survey included an open-ended question on “What can College do to improve well-being” which allowed participants to provide free-text responses to generate more concrete and comprehensive suggestions about possible measures to improve the well-being of OB-GYN doctors.

### 4.5 Limitations

First, as the participation in the survey was voluntary, doctors who were more prone to burnout or mental health issues might have been either more motivated or less inclined to participate, introducing potential self-selection bias and leading to over- or under-estimation of the true prevalence of distress. Secondly, some important potential confounders such as substance and alcohol use and pre-existing psychological conditions were not assessed, which might have influenced the observed associations between occupational factors and mental health outcomes. Thirdly, the cross-sectional design does not allow establishment of causal relationships or the identification of risk factors contributing to the development of burnout, depression or anxiety. This also limited the assessment of long-term effects of interventions. Future research could consider long-term follow-up and evaluate the effectiveness of the interventions.

## 5 Conclusion

The present study showed that OB-GYN doctors in Hong Kong had a high level of burnout, depression, suicidal ideation and anxiety especially among residents-in-training and doctors working for more than 50 h a week. A substantial proportion of the respondents were dissatisfied with their well-being status. The most cited sources of stress were work demands, followed by working hours and on-call duties. Targeted strategies dealing with these stressors are essential to safeguard the well-being of the medical workforce and maintain the provision of high-quality healthcare in Hong Kong.

## Data Availability

The datasets presented in this study can be found in online repositories. The names of the repository/repositories and accession number(s) can be found in the article/supplementary material.

## Footnotes

1
www.raosoft.com/samplesize.html
